# Exploring the Alterations in the Distribution of Neural Network Weights in Dementia Due to Alzheimer’s Disease

**DOI:** 10.3390/e23050500

**Published:** 2021-04-22

**Authors:** Marcos Revilla-Vallejo, Jesús Poza, Javier Gomez-Pilar, Roberto Hornero, Miguel Ángel Tola-Arribas, Mónica Cano, Carlos Gómez

**Affiliations:** 1 Biomedical Engineering Group, E.T.S.I. de Telecomunicación, University of Valladolid, 47011 Valladolid, Spain; jesus.poza@tel.uva.es (J.P.); javier.gomez@gib.tel.uva.es (J.G.-P.); roberto.hornero@tel.uva.es (R.H.); carlos.gomez@tel.uva.es (C.G.); 2 Centro de Investigación Biomédica en Red en Bioingeniería, Biomateriales y Nanomedicina, (CIBER-BBN), 28029 Madrid, Spain; mtola.nrl@gmail.com; 3 IMUVA, Instituto de Investigación en Matemáticas, University of Valladolid, 47011 Valladolid, Spain; 4 Department of Neurology, Río Hortega University Hospital, 47012 Valladolid, Spain; 5 Department of Clinical Neurophysiology, Río Hortega University Hospital, 47012 Valladolid, Spain; mcanopo@saludcastillayleon.es

**Keywords:** Alzheimer’s disease (AD), mild cognitive impairment (MCI), electroencephalogram (EEG), functional neural network, network weights distribution, Shannon entropy (SE)

## Abstract

Alzheimer’s disease (AD) is a neurodegenerative disorder which has become an outstanding social problem. The main objective of this study was to evaluate the alterations that dementia due to AD elicits in the distribution of functional network weights. Functional connectivity networks were obtained using the orthogonalized Amplitude Envelope Correlation (AEC), computed from source-reconstructed resting-state eletroencephalographic (EEG) data in a population formed by 45 cognitive healthy elderly controls, 69 mild cognitive impaired (MCI) patients and 81 AD patients. Our results indicated that AD induces a progressive alteration of network weights distribution; specifically, the Shannon entropy (SE) of the weights distribution showed statistically significant between-group differences (*p* < 0.05, Kruskal-Wallis test, False Discovery Rate corrected). Furthermore, an in-depth analysis of network weights distributions was performed in delta, alpha, and beta-1 frequency bands to discriminate the weight ranges showing statistical differences in SE. Our results showed that lower and higher weights were more affected by the disease, whereas mid-range connections remained unchanged. These findings support the importance of performing detailed analyses of the network weights distribution to further understand the impact of AD progression on functional brain activity.

## 1. Introduction

Alzheimer’s disease (AD) is a neurodegenerative disorder, which causes a significant decline in cognitive function and abnormal behavioral patterns [1]. The increase in life expectancy that characterizes occidental societies has led AD to experiment a significant growth and to become an important social problem [1,2]. It is expected that by mid-century, people with dementia in European Union countries will increase to 16.3 millions, nearly 3.28% of total European Union population [3]. In almost all previous figures, dementia due to AD has a key role [4].

Characteristic physiological changes of AD include the accumulation of senile plaques and neurofibrillary tangles in the brain. Brain dysfunction, loss of synapses, and eventual neuronal death are also associated with AD cognitive decline [5]. The first traces of abnormal protein accumulation begin to appear approximately twenty years before the initial symptoms [1]. Clinically, progressive cognitive decline stands out, characterized by memory loss and a greater effort in brain processes that require planning, reasoning, and problem solving [5]. A prodromal stage, called mild cognitive impairment (MCI), is usually considered in AD progression. MCI is typically addressed as an intermediate stage between normal ageing and dementia, but not always a patient with MCI later develops dementia [1]. The main symptom of MCI subjects is memory loss, while the other cognitive domains usually remain intact [6,7].

AD diagnosis involves a wide variety of tests, which makes it complex and highly dependent on the neurologist who carries out the evaluation [2]. The advances in the knowledge of the disease have caused the revision of clinical diagnosis criteria established by the National Institute of Neurological and Communicative Disorders and Stroke, Alzheimer‘s Disease and Related Disorders (NINCDS-ADRDA) in 1984, leading to National Institute on Aging and Alzheimer’s Association (NIA-AA) diagnosis guidelines, used nowadays [8]. The currenty used AD classification schema is the Amyloid/Tau/Neurodegeneration (A/T/N) [9]. This schema uses biomarkers for beta-amyloid and tau proteins, and for neurodegeneration, obtained from cerebral spinal fluid, positron emission tomography and structural magnetic resonance, to classificate AD patients [9]. The A/T/N biomarkers and the clinical evaluation with cognitive and functional tests, such as Mini-Mental State Examination (MMSE), constitute the diagnosis framework [1,2]. New biomarkers coming from non-invasiveness techniques, like electroencephalogram (EEG) and magnetoencefalogram (MEG), are being explored nowadays and enable a glimpse of the ultimate goal: an early, accurate, and more afordable diagnosis [2,10,11].

Biomedical signals analyses have become an important tool for diagnosing health problems [12]. As stated before, EEG could be very useful to understand brain function and to help in AD diagnosis [2]. EEG records the oscillations of cerebral electrical potentials through electrodes placed on the scalp [13,14]. The recordings obtained using this technique can be used to generate patterns of functional connectivity, which summarize the interactions between neuronal pools. These interactions reflect processing and transmission of brain information [15,16]. Hence, the characterization of the properties of functional brain networks, for instance in terms of segregation, integration, regularity, or complexity, is of paramount importance to understand how neurodegenerative mechanisms involved in diseases as AD disturb higher cognitive functions [16,17].

Previous studies have suggested that entropy is a powerful tool for the quantification of brain function and its processing capacity. Specifically, several papers reveal that the use of entropy measures to analyze the brain regional interactivity could be of paramount importance to understand the changes provoked by brain aging, the altered states of consciousness, and the brain networks ability to process information [18,19,20]. This result supports the use of entropy-based measures to characterize brain alterations caused by neurodegenerative disorders as AD. In fact, some studies characterized the alterations of both local activation patterns and functional connectivity in neural networks [17,18,21,22,23,24]. Other studies have applied entropy to evaluate the hypothesis that neurocognitive systems maintain a dynamic state of balance between order and chaos in terms of dynamics of neural dynamics and functional connectivity, which can be altered with some disorders [19]. In AD, entropy measures have been used to explore the alterations in individual EEG signals, as well as in network properties [7,25,26,27]. Approximate entropy has been used to characterize the irregularity of the EEG [27]. The analysis of coupling parameters in resting-state EEG recordings using cross-entropy metrics have also been helpful to achieve an accurate characterization of the underlying neural dynamics in AD [28]. Moreover, complexity of EEG signals has been evaluated using entropy measures, suggesting an abnormal complexity profile related with AD severity [29]. EEG signal alterations in MCI patients have been also studied using entropic measures, showing their potential to characterize the whole AD continuum [30]. On the other hand, network complexity reflects the capacity of the brain for adaptation and its ability to process information [18,31]. For example, Shannon graph complexity, based on Shannon entropy (SE), captures the interplay between the information and the order of a brain network, and suggested that in complex brain disorders, the brain dynamic reorganization of the neural network is altered [17,32]. Previous works have also suggested that AD affects connectivity patterns in neural networks and, as a consequence, network weights are disturbed [33,34,35]. However, the direct use of SE to characterize networks weights distribution has rather been unexplored, to the best of our knowledge, and may provide a comprehensive understanding of the functional neural network behavior under alterations induced by AD. Hence, the main novelty of this study is the direct use of SE to characterize the alterations caused by AD progression in functional neural network weights distribution. The weights distribution determines to a large extent the network characteristics [16], both those based specifically on the weights of the network and those based in the topology in a weighted network. Thus, the evaluation of the alterations in the network weights distribution could also be helpful to understand the changes in network properties caused by AD. Finally, how the functional brain network is modified along the progression of the disease might reveal details related with the neurodegenerative mechanisms underlying AD.

The hypothesis of this study is that alterations caused by dementia due to AD affect the weights distribution of the functional brain network. We propose to characterize AD effects by applying SE to study the network weights distribution. SE can be useful to summarize intuitively the changes elicited by AD in the network weights distribution. Specifically, in this work the following research questions will be addressed: (i) to what extent does the SE of the network weights distribution reflect brain changes induced by AD progression?; (ii) does the disease affect equally to all network weights or are the alterations focused on specific network connections?

## 2. Materials

### 2.1. Subjects and Variable Collection

The study sample was collected in the Río Hortega University Hospital (Valladolid, Spain). The database consisted of 195 subjects: 45 cognitively healthy controls, 69 patients with MCI due to AD, and 81 patients with dementia due to AD. The groups were formed according the NIA-AA diagnosis criteria [8]. Variable collection was carried out according to the Code of Ethics of the World Medical Association (Declaration of Helsinki) and was approved by the Ethical Committee from the Río Hortega University Hospital (project identification code: 36/2014/02). All subjects, or their relatives in case of incapacity, gave explicit written consent to participate in the study. The written consent includes the purpose of the research, the estimated duration, a comprehensive description of the followed procedures, and a clear indication that the participation is voluntary. The socio-demographic data of each group is detailed in Table 1.

### 2.2. EEG Acquisition and Preprocessing

To acquire the EEG recordings, a 19-channel EEG system (XLTEK, Natus Medical, Oakville, ON, Canada) was used at the Department of Clinical Neurophysiology of Río Hortega University Hospital. The electrodes positioning followed the specifications of the international 10–20 system. In particular the brain activity was recorded from electrodes: Fp1, Fp2, Fz, F3, F4, F7, F8, Cz, C3, C4, T3, T4, T5, T6, Pz, P3, P4, O1 and O2, with common average referencing (CAR). The sampling frequency was 200 Hz. During EEG acquisition, the subjects were asked to stay seated, in resting state and with eyes closed to prevent artifacts. Five minutes of cerebral activity were obtained [36].

The EEG recordings were preprocessed to prepare them for the following analyses. The steps followed are detailed below [7,37]:

1. Digital filtering with a band-pass Finite Impulse Response (FIR) filter, employing a Hamming window in the frequency range 1–70 Hz.
2. Digital filtering with a band-stop FIR filter to remove power line interference at 50 Hz.
3. Independent Component Analysis (ICA) to minimize ocular and muscular artifact-related components.
4. Selection of 5-s artifact-free trials by visual inspection. First twenty artifact-free trials of each subject are included in posterior analyses to have sufficient information and to avoid the influence of different number of artifact-free trials between subjects.

## 3. Methods

### 3.1. Source Reconstruction: sLORETA

Some effects related to sensor-level signals, such as volume conduction or field dispersion, take place and influence the brain activity recorded by EEG. They lead to distorsion and could cause the appearance of unwanted effects in posterior analyses [38,39]. To avoid the limitations induced by the aforementioned effects, the estimation of the original neural generators of acquired EEG activity was carried out. In this study, the standardized low-resolution brain electromagnetic tomography (sLORETA) was used [40]. Its main characteristic is the limitation of the number of possible solutions to the location problem taking into account the synchronization between nearby neurons [40]. The sLORETA algorithm could be freely downloaded from Brainstorm (http://neuroimage.usc.edu/brainstorm, accessed on 10 April 2021) [41]. A comprehensive explanation of the algorithm is available in [40].

The ICBM152 template from the Montreal Neurological Institute, based on 152 magnetic resonances, was used to locate the neural sources [37,42,43]. From this template, a model with 15,000 sources was obtained. By this way, 15,000 source-reconstructed EEG time series were available. Finally, using OpenMEEG software [44], the time series were projected into 68 cortical regions of interest (ROIs) through the Desikan—Killiany atlas [45]. In short, the signals acquired at sensor-level are mapped into 68 ROIs. Henceforth, source-level EEG time series were used.

### 3.2. Connectivity Estimation

Characterizing the complex interactions between cortical regions is of paramount importance to understand transmission and processing of neural information. Different functional connectivity measures have been developed in recent years to describe how brain regions comunicate [46,47] and have been repeatedly used to study the underlying brain mechanisms associated to different neurodegenerative disorders [36,48]. Functional connectivity corresponds to magnitudes of temporal relationships and/or synchrony between time series [16]. Based on previous results [49,50], we used in this study the amplitude envelope correlation (AEC), a functional connectivity metric that estimates the correlation of a pair of signals using their amplitudes [51]. It is important to note that the choice of one connectivity metric or another can highly influence the posterior results [50]. Moreover, many connectivity metrics are sensitive to leakage; hence it is usually required to apply a volume conduction correction [47]. In our study, time series were firstly orthogonalized trial by trial, before computing the AEC. The orthogonalization technique consists on performing a pairwise linear regression between a seed and a test signal to reduce leakage [47]. AEC orthogonalized (i.e., AEC with leakage correction) was obtained after the orthogonalization process. Previous research has shown that AEC with leakage correction is a connectivity metric reproducible and valid in terms of influencing factors, correlation with disease severity, and preferable properties (i.e., correction for volume conduction) in alpha and beta bands, when compared to coherence, imaginary coherence, phase lag index, weighted phaase lag index, and phase locking value [50,52]. Alpha and beta bands have been suggested in previous studies as especially relevant in AD progression [53].Thus, AEC orthogonalized is a robust metric to characterize functional connections and their alterations in AD, while remaining opening the possibility of other measures to assess the functional interactions from different perspectives. After the orthogonalization, signals envelopes were calculated by the Hilbert transform. To evaluate the neural coupling, the correlation between envelopes was quantified by means of the Pearson correlation coefficient [15,54].

Once the functional connectivity patterns were computed, a network with 68 nodes, corresponding to the 68 ROIs, was obtained for each trial of each subject. The connections between the nodes were characterized by the AEC values, which constitute the network weights. In short, a connectivity (adjacency) matrix, whose rows and columns denote the nodes and whose entries denote the values of the connections between related nodes, was obtained [16]. The previously described connectivity estimation was done separately in each one of the conventional EEG frecuency bands: delta (δ, 1–4 Hz), theta (θ, 4–8 Hz), alpha (α, 8–13 Hz), beta-1 (β1, 13–19 Hz), beta-2 (β2, 19–30 Hz), and gamma (γ, 30–70 Hz) [49]. Hence, for each trial of each subject, six connectivity matrices were available for the subsequent analyses: ultimately, a 6 × 68 × 68 × 20 matrix for each subject. The first dimension was the six conventional frequency bands, the subsequent two were the 68 ROIs, whereas the last one was each one of the first twenty artifact-free trials.

### 3.3. Characterization of Network Weights Distribution

In order to characterize the network structure, in a first step, the relative probability histogram of network weights was built [55]. The SE of the network weights distribution was computed from the relative probability histogram. Connectivity values, i.e., network weights, vary between zero and one [16]; therefore, the histogram range was [0, 1]. Each bin of the histogram means the relative probability of network weights with values in the range enclosed by bin edges. The total sum of histogram values was one [55]. In order to create the histogram, the number of bins must be chosen, with the bin width as a dependent variable. It is important to note that these values (i.e., number of bins and width of each bin) must be equal for all subjects, trials, and bands that integrate the database. Thus, the obtained SE values can be directly compared. Different rules have been proposed to estimate the optimal number of bins: Scott, Freedman-Diaconis, Integers, Sturges and Square Root [55]. For the connectivity matrix of each trial and subject, the optimal number of bins was estimated according to all previous mentioned rules. Once these values were obtained, the median level along trials and subjects was computed for each frequency band. The selected number of bins was the highest one, thus allowing to maximize the precision in the SE calculation [32]. This value was 40, obtained in gamma band with the Freedman-Diaconis rule. Hence, giving the histogram range, [0, 1], bin width was set at 0.025.

**Figure 1:**
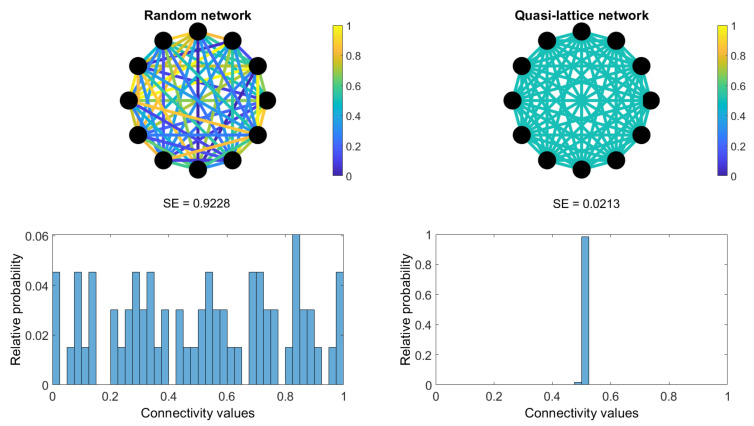
Example for two networks for 12 nodes (**top**) and their weights histograms (**bottom**). The network at the left represents a random network (Erdös-Rényi network, weights uniformely distributed between 0 and 1), while the network at the right is a quasi-lattice network (all the weights with the same value except one). Shannon Entropy (SE) values corresponding to each network are shown in the middle.

Once the number of bins required to estimate the histogram is fixed to 40, the normalized histogram of network weights was estimated for each frequency band, trial, and subject. The estimated network weights distribution is denoted by *q*. At this point, *SE* was computed as [32]:

(1) $$SE=\frac{-1}{\log_{2}N}\sum_{i=1}^{N}\cdot \frac{q}{Q}\cdot \log_{2}\frac{q}{Q}\;,$$

where *Q* is the total sum of relative probability values in each case ($Q=\sum_{i=1}^{N}$*q*) and $\log_{2}N$ is a normalization factor to ensure 0 ≤ *SE* ≤ 1, being *N* the number of elements of the histogram, i.e., number of histogram bins. Finally, to obtain a single *SE* value for each subject at each band, *SE* values across all trials were averaged. Figure 1 shows an example of *SE* behavior. It yields maximum values, SE≈1, when the network weights have a uniform distribution (such a network with highly varied weights, i.e., random network with uniform distribution). In contrast, *SE* yields minimum values, SE≈0, when the network weights have not a uniform distribution (such a network with equally valued weights, i.e., lattice network).

### 3.4. Bin-by-Bin Analysis

Once SE values were computed, histogram values of the trials were averaged to obtain a unique histogram for each subject and frequency band. The purpose of this analysis was to evaluate which particular range of connections of the network weights distribution showed differences between groups. Each histogram value corresponds to network weights in a specific range (i.e., for the first histogram bin, the value corresponded to the relative probability of network weights between 0 and 0.025). Once these vectors were available, it was possible to analyze whether the histograms exhibited statistical differences at each bin.

### 3.5. Statistical Analyses

In a first step, normality of SE values was assessed with the Lilliefors test, while homoscedasticity was evaluated using the Levene test. The obtained results showed that SE values did not meet parametric test conditions. Therefore, non-parametric tests were henceforth used. Kruskal-Wallis test was applied to detect global interactions in SE values at each frequency band. In addition, Mann-Whitney *U*-tests were conducted to assess statistically significant pairwise between-group differences (control group vs. MCI group, MCI group vs. AD group, and control group vs. AD group). Moreover, to evaluate statistical differences at each histogram bin, a Kruskal-Wallis test was carried out. The input values of the test were the relative probability vectors obtained for network weights distributions of each subject and band. Using this procedure, global interactions between the three groups at specific connectivity ranges can be explored. False Discovery Rate (FDR) correction was used to control for type I error [56]. Signal processing and statistical analyses were performed using MATLAB (version R2020b, Mathworks, Natick, MA, USA).

## 4. Results

### 4.1. Socio-Demographic and Clinical Data Analyses

Socio-demographic and clinical data can act as confounding factors. To assess their effects, statistical analyses were initially conducted. Groups showed statistically significant differences by age ($\chi^{2}$(2) = 20.69, *p* < 0.01, Kruskal-Wallis test) and education level ($\chi^{2}$(2) = 15.03, *p* < 0.01, Chi-squared test). No differences were observed by sex ($\chi^{2}$(2) = 1.72, *p* > 0.05, Chi-squared test). MMSE values were lower in MCI and AD groups compared to cognitively healthy control group ($\chi^{2}$(2) = 135.4, *p* < 0.01, Kruskal-Wallis test).

To test whether the differences in age and education level could have an effect in between-group comparisons, Mann-Whitney *U*-tests were performed [49]. Statistically significant differences in age were found in the comparison between MCI and AD groups and between control and AD groups (*p* < 0.05, Mann-Whitney *U*-test). In order to assess the effect on SE values of between-group age differences, a Spearman’s bivariate correlation test was performed [57]. The results showed that age distribution and SE values did not show a statistically significant correlation (*p* > 0.05, Spearman’s bivariate correlation test, FDR corrected). Finally, no statistical differences were found in SE values in any between-group comparisons using education level as grouping variable (*p* > 0.05, Mann-Whitney *U*-test).

### 4.2. Shannon Entropy of Network Weights Distribution

**Figure 2:**
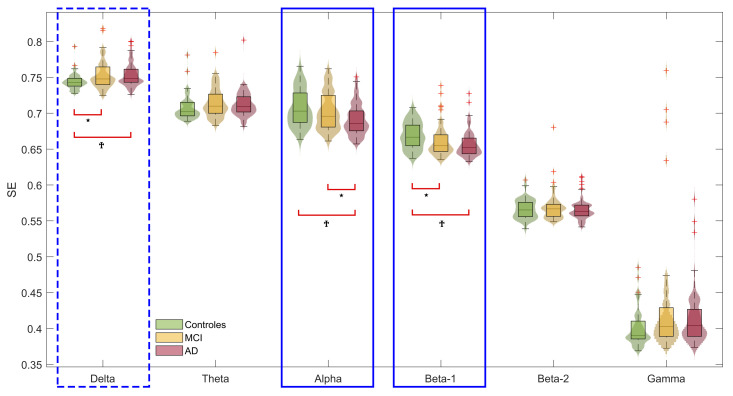
Box plots depicting SE values of network weights distribution in each frecuency band under study. For each box plot, the lower bar represents the first quartile, the middle bar reflects the median and the upper bar marks the third quartile. The whiskers mark the most extrem points not considered outliers and the red crosses represent the outliers. Statistically significant between-group differences are marked with discontinuous blue rectangles (*p* < 0.05, Kruskal-Wallis test, False Discovery Rate (FDR) corrected) and with continuous blue rectangles (*p* < 0.01, Kruskal-Wallis test, FDR corrected). Statistically significant between-group pairwise comparisons are marked with red brackets (*: *p* < 0.05, Mann-Whitney *U*-tests, FDR corrected; †: *p* < 0.01, Mann-Whitney *U*-tests, FDR corrected).

SE values of network weights distribution were computed for all the subjects and frequency bands. Figure 2 summarizes the results.

Statistically significant differences between the three groups were found in delta, alpha, and beta-1 bands (*p* < 0.05 in delta band; *p* < 0.01 in alpha and beta-1 bands; Kruskal-Wallis test, FDR corrected). In the case of delta band, AD group showed higher SE values than MCI and control groups. However, in alpha and beta-1 bands, the behavior was the opposite: AD group showed a lower SE than control and MCI groups. As showed in Figure 2, control and AD groups exhibited statistically significant differences in delta, alpha, and beta-1 bands (*p* < 0.01, Mann-Whiney *U*-test, FDR corrected). Control and MCI groups showed statistically significant differences in delta and beta-1 bands (*p* < 0.05, Mann-Whitney *U*-test, FDR corrected). MCI and AD groups only showed differences in alpha band (*p* < 0.05, Mann-Whitney *U*-test, FDR corrected).

### 4.3. Histogram of Network Weights Analysis

**Figure 3:**
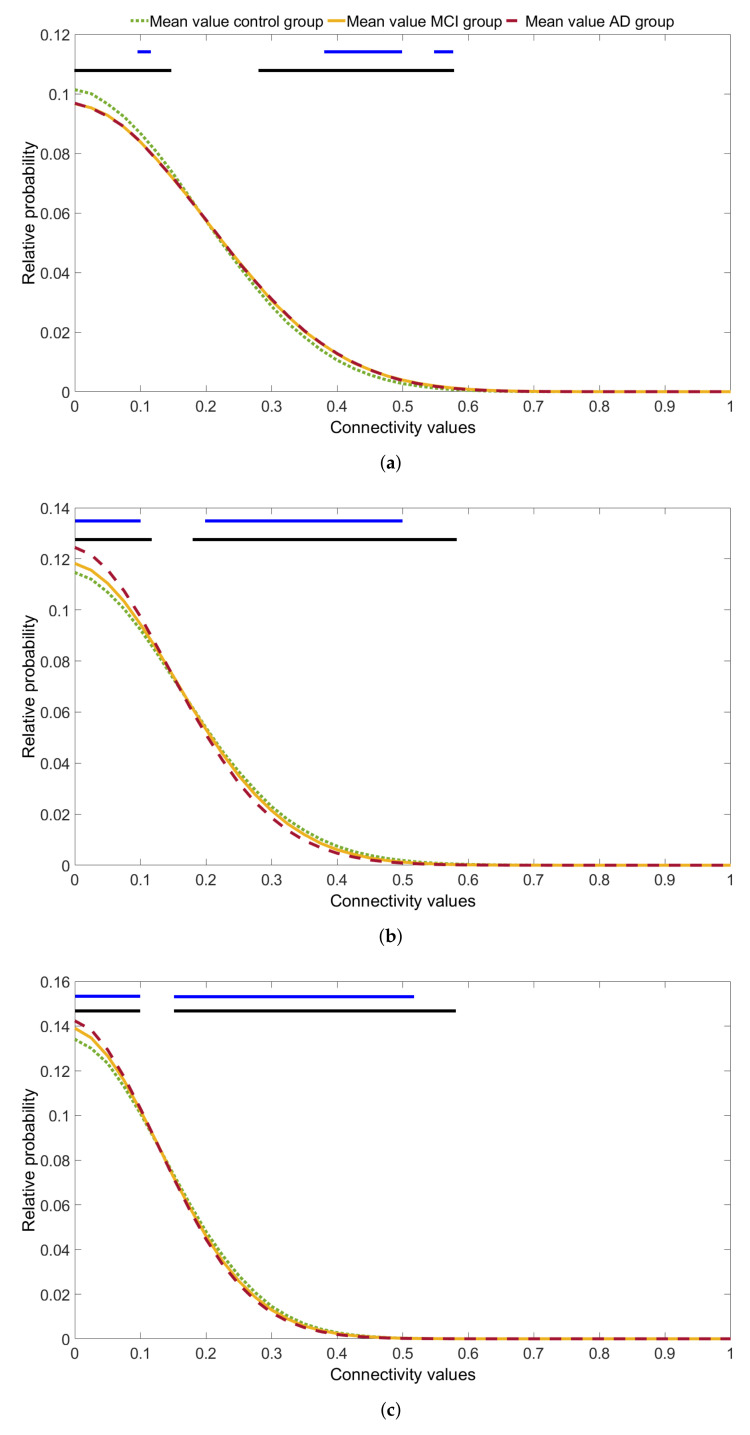
Average histograms of network weights distribution for each group. The upper lines denote statistically significant between-groups differences (blue lines: *p* < 0.01, Kruskal-Wallis test, FDR corrected *p*-values; black lines: *p* < 0.05, Kruskal-Wallis test, FDR corrected *p*-values). (**a**) Delta band. (**b**) Alpha band. (**c**) Beta-1 band.

In a second step, the distribution of histogram values was in-depth analyzed at each bin. The evaluation of statistical differences for each histogram bin allowed us to establish the connectivity ranges where the groups presented statistically significant differences. Moreover, this analysis is useful to evaluate how AD affects specific functional neural network weights. In order to reduce type I errors, this analysis was performed only in frequency bands in which statistically significant differences in the global comparison between groups were observed, i.e.,: delta, alpha, and beta-1. Figure 3 shows the evolution of average histogram values for each group.

The observed evolution was quite similar in the three frequency bands. Most of network weights have values around 0.1. The number of weights decreases as the connectivity values increase. Statistically significant differences were found in the ranges between 0 and 0.1, as well as between 0.3 and 0.6 (*p* < 0.05, Kruskal-Wallis test, FDR corrected). The interval [0.1, 0.25], aproximately, did not show differences in any band. In fact, a dichotomy between low (i.e., [0, 0.1]) and high ([0.3, 0.6]) network weights was clearly discernible.

As seen in Section 4.2, delta band showed non-statistically significant differences in the comparison between MCI and AD groups. However, the comparison between control and AD groups showed interesting differences. The former has more connections with low network weights, while the latest has more high network weights. On the other hand, alpha and beta-1 bands exhibited a different pattern. Alpha band presented stronger differences than the other bands. It can also be observed that cognitively healthy control group had more connections with high network weights than MCI and AD groups. Newly, it is possible to observe the distinction between low frequency bands (delta) and higher ones (alpha and beta-1).

Finally, looking at Figure 3a–c, it can be observed that as frequency increases (delta, alpha, and beta-1 bands) the relative probability of lower connectivity values is higher, while the relative probability of higher ones decreases.

## 5. Discussion

In the present study, SE values of network weights distribution from cognitively healthy elderly individuals and patients with MCI and dementia due to AD were analyzed. To this end, relative probability histograms were used. Furthermore, histogram values were analyzed bin by bin to evaluate which network weights experimented more changes among groups. Our findings suggest that: (i) MCI and AD induce frequency-dependent alterations in network weights distribution which are reflected in its SE values; (ii) the evaluation of the effects of MCI and AD in specific network weights suggests an interesting dichotomy between low and high connections.

### 5.1. Shannon Entropy Is Useful to Characterize Network Weights Distribution

As stated, the hypothesis of this study was that dementia due to AD disrupts the functional brain network by altering the network weights distribution. SE showed global statistically significant differences between the three groups in delta, alpha, and beta-1 bands (Figure 2). Analyzing in further detail the values, the patterns between groups change in delta with respect to alpha and beta-1 bands. Controls exhibited lower SE values in delta band, while SE values were higher in alpha and beta-1 bands. Previous studies have reported differences between low and high conventional frequency bands. Koelewijn et al. (2017) observed that whole-brain assessments showed that disrupted regional oscillatory envelope amplitude and connectivity in alpha and beta bands play a key role in AD [53]. Their findings agree with our results, since alpha and beta-1 bands are tightly aligned and, in both bands, MCI and AD patients showed lower SE values. Another study carried out by Babiloni et al. concluded that MCI and AD populations showed abnormalities at specific frequency bands, specifically below 12 Hz, strongly associated with altered functional and effective EEG connectivity [58]. Connectivity values, that is network weights, reflect the alterations due to MCI and AD. Therefore, SE computed using network weights distribution also reflects these alterations. The SE decrease suggests functional connectivity networks with less varied connections, probably related with the neuronal death and the deficit in synapses caused by AD progression [7,59]. De Haan et al. demonstrated that a loss of communication between different functional brain regions reflected cognitive decline caused by AD [60]. This loss of communication can be quantified in functional neural networks as a decrease of integration, namely, a reduction in network ability to combine specialized information from distributed regions [16]. Higher network weights contribute to higher integration. Therefore, higher SE values of the network weights distribution in elderly healthy control group suggest the presence of network weights with higher values (i.e., higher connectivity) and a significant integration. Thus, our findings lead up to a possible integration decrease as dementia due to AD progresses, which has been also suggested in previous studies [16,46,48].

In line with the previous ideas, the debate around the balance between integration and segregation network properties arises [61]. Preceding studies suggested that memory processing in MCI subjects is associated with a tendency toward random structure, which breaks the balance between integration and segregation [62]. Buldú et al. suggested that, given the high rate of conversion from MCI to AD, the analysis of functional networks and the balance between the aforementioned network properties could be useful for an early detection of dementia [62]. We have already discussed the integration decrease as AD advances, reflected in the reduced SE values. Nonetheless, the decrease in SE values could also reveal the mentioned imbalance. When the network ability to use and combine information from different regions decreases (loss of integration), network segregation (i.e., network ability for specialized processing to occur within densely interconnected groups of brain regions [16]) acquires a more important dimension. Lower connectivity values (i.e., lower network weights) usually involve largest distances in the network; hence, they facilitate specialized processing, concentrated in specific brain regions. Therefore, MCI and AD functional networks should be composed by connections whose weights distribution has lower SE values, as it could be seen in Figure 2.

An additional worthwhile fact is the statistically significant differences between healthy controls and, both, MCI (*p* < 0.05, Mann-Whitney *U*-tests, FDR corrected) and AD (*p* < 0.01, Mann-Whitney *U*-tests, FDR corrected) patients groups. SE values allow to distinguish the non-pathological group with respect to pathological groups in delta and beta-1 bands. In alpha band this distinction is not possible; however, in this band MCI and AD groups showed statistically significant differences. This change could be related with previous findings which suggest that, in healthy aging, alpha oscillations decrease in some brain areas, but this decrease is higher in MCI and AD patients [53,63]. In particular, MCI is usually considered as an intermediate state between AD and normal aging. Thus, the decrease in alpha band oscillations could be responsible of the differentation between SE values of MCI and AD patients in this band. The variation on oscillations, that is EEG rhythms, affects local connectivity values (i.e., local sincronization) and, consequently, SE values computed from connectivity.

The use of entropy-based metrics to characterize functional network weights distribution is rather unexplored in AD. The connections found reveal interesting issues concerning to AD. Differences between pathological and non-pathological groups might show, first, the importance of network weights distribution to understand AD progression. Secondly, SE can be useful to assess the balance and imbalance of the network. Network properties (i.e., integration and segregation) are tightly related with these concepts. SE values next to 1 reflect a network with weights uniformely distributed; whereas, SE values next to 0 reflect a network with equally valued weights, less variety. Figure 2 shows, in delta, higher SE values in AD group; meanwhile, it is also possible to observe that alpha and beta-1 frequency bands have lower SE values in AD patients. Conventional frequency bands have different responses to AD alterations [53]. Delta band results suggest functional network weights with more uniform distributions as AD progresses. Instead, alpha and beta-1 bands suggest functional network weights with less uniform distributions as AD progresses, more similar weights values. In fact, we could see the previously mentioned dichotomy between frequency bands where statistically significant differences were found. Therefore, the AD progression could be related with a decompensation of integration and segregation network properties. This decompensation could appear by disturbing the network in a way in which less varied weights exist (SE close to 0), or by modifying connections to obtain a network with highly different weights (SE close to 1).

### 5.2. MCI and AD Modify the Distribution of Low and High Connectivity Values

We have already discussed the usefulness of SE, applied to the brain network weights distribution, to characterize the alterations in AD progression from a global perspective. However, we perfomed an in-depth bin-by-bin analysis of the histogram of network weights to evaluate the specific ranges of connectivity values that differed between groups. Figure 3 shows a dichotomy between low and high connectivity values (common to the delta, alpha, and beta-1). In these ranges, specifically in [0, 0.1] and [0.3, 0.6], statistically significant differences between groups were reached (*p*-value < 0.05, Kruskal-Wallis test, FDR corrected), whereas mid-range connections remain almost unaltered. In fact, the most extreme connections, specifically the higher ones, are those critical to network performance, because changes in their number drastically modify network properties [64]. AD might reduce brain activity interactions (i.e., functional connectivity) by increasing the number of lower connections and by disminishing weights of higher ones. As we analyzed EEG activity, the reduction in these interactions is in line with previous findings that suggested a complexity loss and an irregularity reduction in individual EEG signals [13,65].

Compared to the whole range of possible connectivity values, the relative probability of low network weights was higher in the three groups under analysis, but cognitively healthy control group showed that network weights with low values were less probable than for MCI and AD groups in alpha and beta-1 frequency bands. Coming back to comments in Section 5.1, lower network weights are associated with a decreased network integration. Therefore, the in-depth histogram analysis supports the global results previously analyzed; likewise, they are in agreement with those reported by previous studies that found a reduced integration in MCI and AD patients’ brain networks [16,46,48]. The decrease in the ability of the brain network to integrate information could be related with an increased difficulty to trasmit neural information between spatially distributed regions. This fact might evidence the network disconnection, typically observed in AD [66]. Numerous studies have addressed this hypothesis and have gather evidence in line with these disconnection hypothesis [66,67,68].

Although the brain conducts specialized processing in specific regions, its global functioning also depends on the correct communication within distinct areas [16]. Ultimately, it is referred to the balance between integration and segregation of brain network that we have previously mentioned [16,61,62]. Connections with high values foster brain integration and segregation. Consequently, a network in which this balance has led to the optimal end, should have more connections with high values and less with low values. Regarding to Figure 3, functional networks of cognitively healthy controls behave in this way, specifically in alpha and beta-1 bands. The relative probability of low connections was lower in these networks than in those observed for MCI and AD. Moreover, the relative probability of network weights with higher values increased. However, in MCI, network connections with low values were more likely, just the opposite that for the high ones. In AD networks, this behavior was more evident. In sum, these results suggest that an imbalance between integration and segregation occurs along dementia progression and supports the results obtained by previous research [61,62]. Delta band behaved differently to alpha and beta-1 bands (Figure 3). Previous studies addressed these differences and argued that low frequency bands have different responses to those showed by higher frequency ones [28]. In addition, alpha and beta bands have been found to be particularly relevant in AD progression [53].

### 5.3. Limitations and Future Research Lines

Firstly, EEG recordings allow to characterize brain activity with high temporal resolution. However, their spatial resolution is lower than the obtained with techniques such as magnetic resonance imaging. For this reason, the use of EEG recordings in combination with techniques with higher spatial resolution could be useful to provide a more comprehensive characterization of the functional neural network and the alterations caused by AD. Secondly, the obtained results are highly dependent on the connectivity metric chosen to characterize the functional brain interactions between the ROIs [47]. For this reason, the application of other approaches to achieve a comprehensive characterization of the functional interactions could be an interesting future step [69]. Thirdly, when we applied SE to network weights distribution, we cannot obtain information about the network topology. Topology and network weights distribution are key network properties; thereby, future analyses should address the effects of topology alterations along AD progression. Fourthly, SE is not sensitive to histograms showing similar shapes, but shifted, which could bias the results. Nevertheless, our bin-to-bin analysis indicate that network weights distribution exhibits a similar shape. It would be interesting that future studies introduce new entropy-based metrics that overcome this issue. Finally, the proposed SE measure does not address more complex network properties, such as robustness, resilience, or plasticity, among others. These properties incorporate a network dynamic response to perturbations. Hence, they could be useful to gain further comprehension on neurodegenerative mechanisms underlying diseases as AD. Nevertheless, an in-depth characterization of the brain network with parameters as the SE is necessary to warrant the reliability of these new measures.

## 6. Conclusions

Our results suggest that the SE of network weights distribution is a useful measure to characterize functional network alterations caused by dementia due to AD. The study of the distribution of network weights plays a key role to undertand the neural mechanisms involved in the disease continuum. Furthermore, our results support the hypothesis of AD as a disconnection syndrome, as well as the importance of the imbalance between network integration and segregation to understand the alterations on functional network elicited by AD. We also showed the interest of an in-depth analysis of connectivity values to evaluate whether all network connections are equally affected by the disease or not. Our findings suggest that lower and higher network weights are more important in the AD continuum than mid-range ones.

## Figures and Tables

**Table 1:** Socio-demographic and clinical data of the population included in the study.

Data	Group		
	Controls	MCI Subjects	AD Patients
Number of subjects	45	69	81
Age (years) (m[IQR]) ${}^{1}$	75.6 [73.88, 78.63]	77.1 [72.25, 80.33]	81.7 [76.25, 83.53]
Sex (M:F) ${}^{2}$	14:31	29:40	34:47
Education level (A:B) ${}^{3}$	17:28	43:26	59:22
MMSE ${}^{4}$ (m[IQR])	29 [28, 30]	27 [26, 28]	21 [18, 24]

^1^ m: median; IQR: interquartile range; ^2^ M: male; F: female; ^3^ A: primary education or below; B: secondary education or above; ^4^ MMSE: Mini-Mental State Examination (range: [0, 30]).

## Data Availability

The datasets analyzed in the study are available from the authors upon reasonable request.

## Abbreviations

The following abbreviations are used in this manuscript:

AEC	Amplitude Envelope Correlation
AD	Alzheimer’s disease
A/T/N	Amyloid/Tau/Neurodegeneration
CAR	Common Average Referencing
EEG	Electroencephalogram
FIR	Finite Impulse Response
FDR	False Discovery Rate
ICA	Independent Component Analysis
MCI	Mild Cognitive Impairment
MEG	Magnetoencephalogram
MMSE	Mini-Mental State Examination
NIA-AA	National Institute on Aging and Alzheimer’s Association
NINCDS-ADRDA	National Institute of Neurological and Communicative Disorders and Stroke,
	Alzheimer’s Disease and Related Disorders
ROI	Region of Interest
sLORETA	Standarized low—resolution brain electromagnetic tomography
SD	Standard deviation
SE	Shannon entropy
